# Improving Corrosion Resistance of Rare Earth Zirconates to Calcium–Magnesium–Alumina–Silicate Molten Salt Through High-Entropy Strategy

**DOI:** 10.3390/ma17246254

**Published:** 2024-12-21

**Authors:** Cong Gui, Zi-Jian Peng, Jun-Teng Yao, Shu-Qi Wang, Zhan-Guo Liu, Ya-Ming Wang, Jia-Hu Ouyang

**Affiliations:** School of Materials Science and Engineering, Harbin Institute of Technology, Harbin 150001, China; 22s009056@stu.hit.edu.cn (C.G.); pengzijianhit@163.com (Z.-J.P.); yaojuntengjyt@163.com (J.-T.Y.); zgliu@hit.edu.cn (Z.-G.L.); wangyaming@hit.edu.cn (Y.-M.W.)

**Keywords:** high-entropy rare earth zirconates, CMAS corrosion behavior, rare earth cations, thermal barrier coatings

## Abstract

The erosion caused by high-temperature calcium–magnesium–alumina–silicate (CMAS) has emerged as a critical impediment to the advancement of thermal barrier coating (TBC). In this study, a series of high-entropy rare earth zirconates, (La_0.2_Sm_0.2_Dy_0.2_Er_0.2_Gd_0.2_)_2_(Zr_1−*x*_Ce*_x_*)_2_O_7_ (*x* = 0, 0.2, 0.4, 0.5) were synthesized through a solid-phase reaction, and their corrosion behavior against CMAS was investigated. Our findings demonstrate that numerous rare earth elements impede element diffusion, facilitate the formation of a compact oxide layer, and effectively hinder CMAS infiltration. Furthermore, rare earth elements with larger ionic radii exhibit enhanced solubility in apatite, whereas those with smaller ionic radii are more readily soluble in ZrO_2_. In general, the utilization of the high-entropy strategy is an effective approach to significantly improving corrosion resistance against CMAS.

## 1. Introduction

Thermal barrier coatings (TBCs) are widely applied to the surface of high-temperature metal components in gas turbines and aviation engines [1,2]. TBCs can effectively isolate high-temperature gas, reduce the surface temperature of superalloy, and extend the service life of components [3,4]. 7–8 wt.% The preferred material in TBCs is Yttria-stabilized Zirconia (YSZ) due to its excellent thermal and mechanical properties [5]. However, it is known that high-temperature service environments (>1200 °C) can cause phase transformations in YSZ coatings (*t*′→*m*), resulting in 3–4% volumetric expansion and TBCs system failure [6]. With the development of high-thrust-ratio gas turbines, the inlet temperature of gas turbines is gradually increasing, posing new challenges for traditional TBC materials. Rare earth zirconates (RE_2_Zr_2_O_7_) possess low thermal conductivity (1.0–1.6 W·m^−1^·K^−1^), high thermal expansion coefficients (9–11 × 10^−6^·K^−1^), good resistance to sintering, and excellent high-temperature stability, making them ideal candidates for new thermal barrier coatings [7,8,9,10].

At the same time, solid particles in the air (such as volcanic ash, sand, dust, etc.) are ingested into the engine along with the intake air. These particles, primarily composed of CaO, MgO, Al_2_O_3_, and SiO_2_ (calcium–magnesium–alumina–silicate, CMAS), melt at high temperatures and deposit on the surface of the TBCs [11,12]. They then penetrate and react chemically with the coating materials, significantly reducing the service life of the coatings [13,14]. Studies have shown that RE_2_Zr_2_O_7_ has better resistance to CMAS corrosion than YSZ [15,16]. The doping strategy and high-entropy strategy have been proven to be effective means to regulate the corrosion resistance of CMAS [17,18]. Deng et al. [19] indicate that during the corrosion process of high-entropy rare earth zirconates (HE-REZ) against CMAS, the larger the radius of the RE^3+^ ions at the A-site, the more likely it is to form apatite, which can slow down the further penetration of CMAS [20]. And rare earth elements with a smaller ionic radius are more likely to participate in stabilizing ZrO_2_ [21,22]. The retarded diffusion effect in high-entropy rare earth zirconates leads to the formation of a fine-grained, dense reaction layer which may increase the layer’s tolerance to cracking during the thermal cycle process of TBCs [23]. This is attributed to the different diffusion rates of various rare earth elements in apatite, resulting in a fine-grained state of the barrier layer, which alleviates thermal mismatch during the reaction [24]. Additionally, increasing the content of the fluorite phase can enhance corrosion resistance against CMAS [25].

A review of previous work reveals that there is relatively little research on high-entropy rare earth zirconates doped at both the A and B sites, as well as studies on their CMAS corrosion behavior. Therefore, this paper primarily focuses on doping La, Sm, Dy, Er, and Gd at the A site and Ce at the B site to design and synthesize high-entropy ceramics ((La_0.2_Sm_0.2_Dy_0.2_Er_0.2_Gd_0.2_)_2_(Zr_1−*x*_Ce*_x_*)_2_O_7_ (*x* = 0, 0.2, 0.4, 0.5); investigating the CMAS corrosion behavior of high-entropy rare earth zirconate ceramics with different compositions at 1300 °C over various reaction times; and exploring the influence mechanism of the high-entropy strategy on CMAS corrosion behavior via a comparison with La_2_Zr_2_O_7_.

## 2. Experimental Procedures

### 2.1. Preparation of Ceramic Sample

The four high-entropy ceramics of (La_0.2_Sm_0.2_Dy_0.2_Er_0.2_Gd_0.2_)_2_(Zr_1−*x*_Ce*_x_*)_2_O_7_ (*x* = 0, 0.2, 0.4, 0.5) and La_2_Zr_2_O_7_ were prepared through a two-step solid-state reaction process. Initially, commercial RE_2_O_3_ (RE = La, Sm, Dy, Er, Gd) (purity 99.9%, Grirem Advanced Materials Co., Ltd. Beijing, China) ZrO_2_ (purity 99.9%, Shanghai St-Nano Science and Technology Co., Ltd. Shanghai, China), and CeO_2_ (purity 99.9%, Grirem Advanced Materials Co., Ltd. Beijing, China) were used as raw materials. These were weighed according to their stoichiometric ratios, mixed with an appropriate amount of anhydrous ethanol, and ball-milled at a speed of 300 rpm for 24 h. The resulting slurry was dried at 60 °C and sieved through a 200-mesh screen to obtain the required powder. The powder was then calcined in a muffle furnace at 1400 °C for 6 h to achieve a solid-state reaction, followed by grinding in a mortar and sieving through a 200-mesh screen. Subsequently, the powder was compacted under a uniaxial pressure of 10 Mpa for 3 min to form green bodies, which were then placed under a cold isostatic press at 200 Mpa for 3 min. Finally, the green bodies were sintered in air at 1650 °C for 10 h to obtain dense ceramics. The ceramic compositions and heat treatment processes are shown in Table 1. The densities of all prepared samples are displayed in Table 2.

### 2.2. Fabrication of CMAS Glass Powder and Isothermal Corrosion

The composition of CMAS powder was 33 mol.% CaO, 9 mol.% MgO, 13 mol.% AlO_1.5_, and 45 mol.% SiO_2_. (Ca_33_Mg_9_Al_13_Si_45_O_151.5_) in this work. First, the raw materials were weighed according to the above ratio; then, the powders of CaO (AR, 99%, Shanghai Macklin Biochemical Technology Co., Ltd., Sanghai, China), MgO (>99%, Shanghai Macklin Biochemical Technology Co., Ltd., Shanghai, China), Al_2_O_3_ (AR, 99%, Shanghai Macklin Biochemical Technology Co., Ltd., Shanghai, China), and SiO_2_ (GR, 98.5%, Shanghai Macklin Biochemical Technology Co., Ltd. Shanghai, China) were placed into a sealed jar. All the powders were thoroughly mixed during ball-milling at a rotation speed of 300 rpm for 24 h. After drying at 60 °C, the powders were heated to 1400 °C for 8 h to obtain the CMAS melt, and then poured it into deionized water to obtain CMAS glass. The fine CMAS powder was obtained through grinding and sieving under a 200-mesh screen.

The prepared bulk ceramic samples were all circular disks with a diameter D of 20 mm and a thickness H of 3 mm. After polishing the ceramic disks to a surface roughness of 1 μm, the CMAS glass powder was spread on the sample surfaces with a concentration of 30 mg/cm^2^. The samples were then transferred to a muffle furnace and subjected to corrosion at 1300 °C for 2, 4, and 8 h.

### 2.3. Material Characterization

The phase structure of the calcined ceramic powder and the bulk ceramic before and after CMAS corrosion was characterized using X-ray diffraction (XRD, Empyrean, PANalytical B.V., Almelo, Netherlands) with a scanning range of 2θ from 10° to 90° and a scanning speed of 10°/min. The melting point was measured from room temperature to 1400 °C using differential scanning calorimetry (DSC; STA 449 C, NETZSCH, Selb, Germany). The cross-sectional morphology and elemental distribution before and after CMAS corrosion were observed using a scanning electron microscope with X-ray energy-dispersive spectroscopy (SEM, SU5000, HITACHI, Tokyo, Japan). The chemical composition of the CMAS glass was determined using an Inductively Coupled Plasma Optical Emission Spectrometer (ICP-OES, Thermo, iCAP 7400, Hanau, Germany).

## 3. Results

### 3.1. Characterization of Ceramic Bulk Materials Prepared by Solid-State Reaction Method

**Figure 1:**
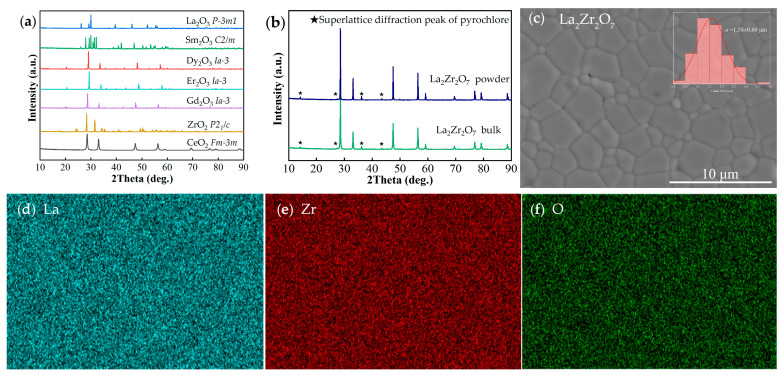
(**a**) XRD patterns of raw material powders; (**b**) XRD patterns of La_2_Zr_2_O_7_ powder and bulk material; (**c**) surface morphology and grain size distribution of bulk La_2_Zr_2_O_7_ prepared by two-step method; (**d**–**f**) element distribution map of bulk La_2_Zr_2_O_7_.

The XRD patterns of the raw material powders, prepared La_2_Zr_2_O_7_ powder, and bulk material are shown in Figure 1a,b. It can be observed that after treatment at 1400 °C for 6 h, La_2_Zr_2_O_7_ powder is transformed into single-phase ceramic powder, and the both powder and bulk materials have distinct superlattice diffraction peaks of the pyrochlore structure with the space group $Fd\bar{3}m$(227) [26]. The XRD diffraction peaks of the bulk material are relatively sharp, and no oxide impurity peaks are observed, which illustrates the successful synthesis of La_2_Zr_2_O_7_. The surface morphology and an element distribution map of the prepared La_2_Zr_2_O_7_ are presented in Figure 1c–f, and it exhibits a fine and dense microstructure, well-distributed elements, a distinct polygonal grain boundary with no evidence of impurity precipitation, and a relatively uniform grain size with an average size of 1.58 ± 0.60 μm.

**Figure 2:**
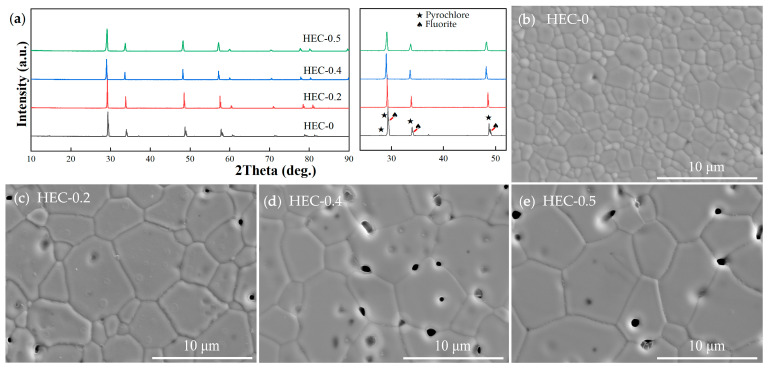
(**a**) XRD patterns of high-entropy ceramics. (**b**–**e**) Surface morphology of high-entropy ceramic series: (**b**) HEC-0; (**c**) HEC-0.2; (**d**) HEC-0.4; (**e**) HEC-0.5.

The XRD pattern and surface morphology of the high-entropy ceramic series are shown in Figure 2. Only diffraction peaks of fluorite, including (111), (200), (220), (311) and (222), can be identified in HEC-0.2 to HEC-0.5, proving that these three high-entropy ceramics exhibit a single fluorite structure [26]. Meanwhile, it can been seen that the sample of HEC-0 reveals two series of diffraction peaks, typical superlattice diffraction peaks of pyrochlore structures and of fluorite structures, and are marked with pentagrams and spades, which indicates that HEC-0 is a pyrochlore–fluorite dual-phase zirconate [27,28]. Dual-phase HEC-0 exhibits a smaller grain size (1.10 ± 0.61 μm) than the rest of the high-entropy ceramic series, and possesses two different sizes. The rest of the high-entropy ceramics (HEC-0.2; HEC-0.4; HEC-0.5) show a similar defective fluorite-type non-uniformly polygonal structure without impurity, with grain sizes of 2.95 ± 1.55 μm, 3.93 ± 1.75 μm, and 4.99 ± 2.51 μm, respectively.

**Figure 3:**
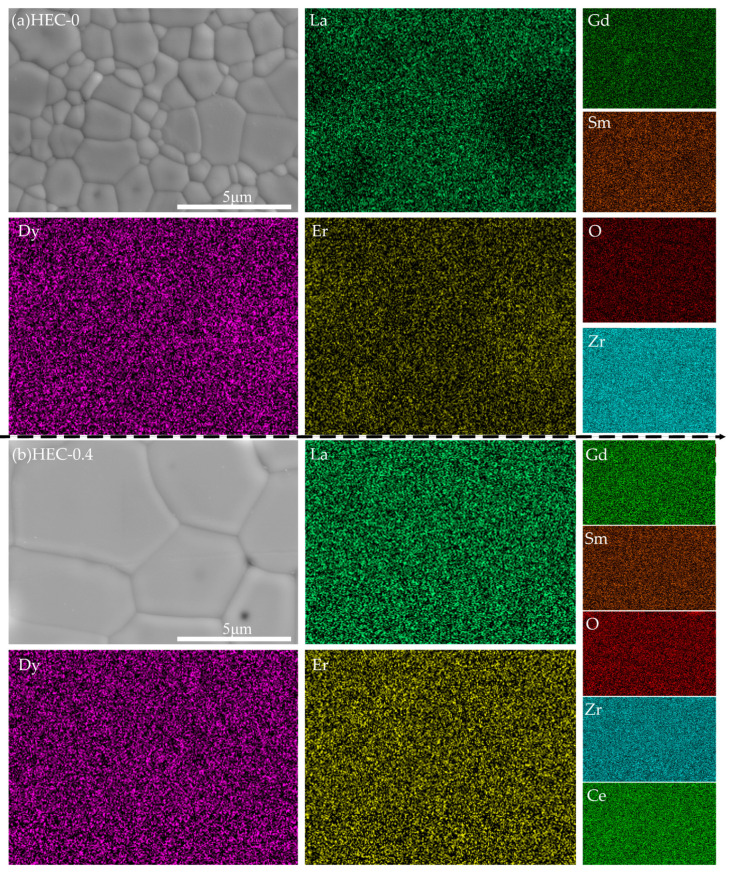
(**a**) Surface morphology and element distribution map of dual-phase HEC-0 ceramic. (**b**) Surface morphology and element distribution map of single-phase HEC-0.4 ceramic.

To further investigate the elemental distribution in high-entropy rare earth zirconates, SEM observations and corresponding element mappings were performed, as shown in Figure 3. A typical defective fluorite-type sample is shown in Figure 3b with relatively larger uniform polygonal grains. The elements of rare earth (La, Ce, Sm, Gd, Dy and Er) and Zr are homogeneously distributed in the HEC-0.4 sample. However, the trend of rare earth element segregation is different in the dual-phase high-entropy rare earth zirconate. For the sample HEC-0, Ce, Sm, Gd, and Zr are relatively uniform in grains with two different sizes, but La with a larger ionic radius is enriched in smaller grains, and Er and Dy with a smaller ionic radius are enriched in larger grains, which exerts a direct influence on the cation radius ratio (*r*_A_/*r*_B_), resulting in the formation of different phase structures. The increase in La concentration leads to a relative increase in *r*_A_/*r*_B_ within the small grains, thereby favoring the formation of a pyrochlore structure, while the larger grains are the opposite. Meanwhile, the mutual diffusion of rare earth elements and the competitive growth of the two phases lead to the finer grain size than single-phase zirconates [27,29,30].

### 3.2. Characterization of CMAS Used in High-Temperature Corrosion Experiments

**Figure 4:**
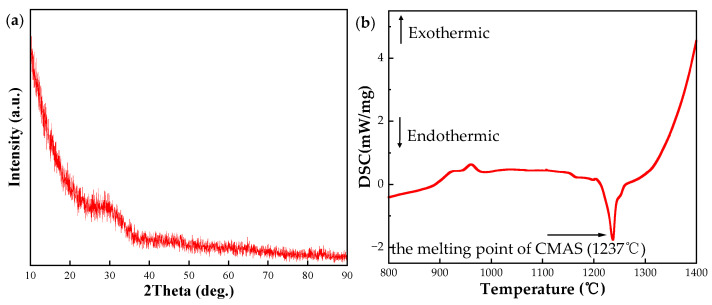
XRD spectrum (**a**) and DSC analysis (**b**) of CMAS glass powder.

The CMAS glass powder was subjected to XRD diffraction and DSC analysis to determine its purity and melting point, with the results shown in Figure 4a,b. The prepared CMAS powder exhibits no characteristic diffraction peaks, proving that it is completely amorphous and no other crystalline phases were precipitated. From the DSC measurement result, the curve drops sharply around 1210 °C and reaches its lowest point at 1237 °C, indicating that the melting point of the CMAS glass is 1237 °C. The melting point is lower than the designed high-temperature corrosion test temperature of 1300 °C, suggesting that the CMAS powder can completely melt, wet, and spread on the bulk surface. Finally, the ICP-OES test indicates that the actual composition of the CMAS glass is 30.2 mol.% CaO-8.9 mol.% MgO-13.5 mol.% AlO_1.5_-47.4 mol.% SiO_2_.

### 3.3. High-Temperature CMAS Corrosion Behavior at 1300 °C

**Figure 5:**
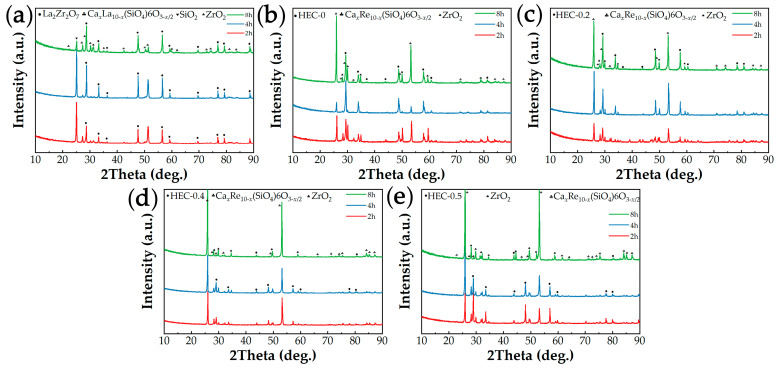
XRD patterns of five rare earth zirconate blocks after CMAS corrosion: (**a**) La_2_Zr_2_O_7_; (**b**) HEC-0; (**c**) HEC-0.2; (**d**) HEC-0.4; (**e**) HEC-0.5.

The XRD patterns of five rare earth zirconate blocks after 2, 4, and 8 h of CMAS corrosion at 1300 °C are illustrated in Figure 5. The XRD peaks of the five rare earth zirconate blocks exhibit remarkable similarity even after undergoing different corrosion durations. In addition to the residual CMAS melt and SiO_2_ on the surface, varying degrees of apatite (Ca*_x_*Re_10−*x*_(SiO_4_)_6_O_3−*x*/2_, 2 ≤ *x* ≤ 6) and ZrO_2_ peaks are observed as the main corrosion products. The peak strength of apatite and zirconia increases with prolonged corrosion time, while the peak shape becomes more pronounced.

**Figure 6:**
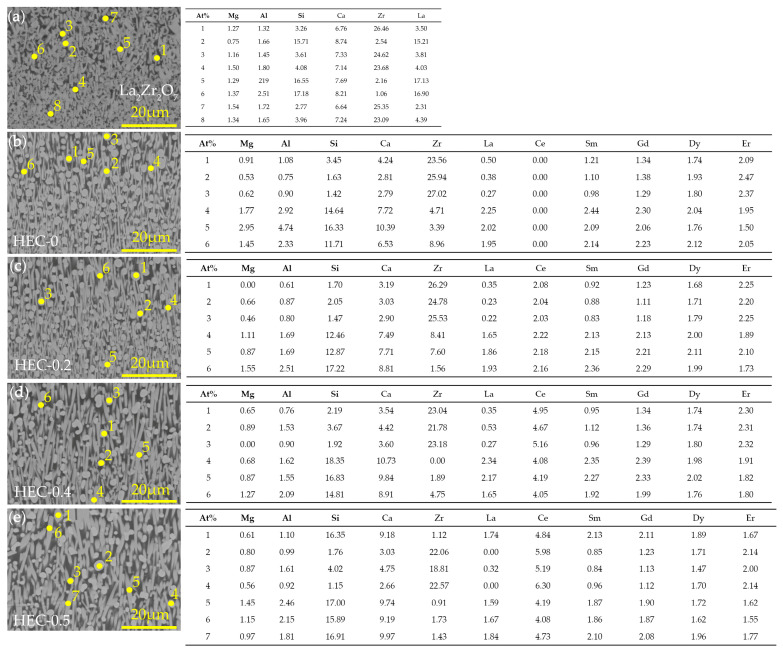
Cross-sectional morphology and semi-quantitative element distribution of five rare earth zirconate blocks after CMAS corrosion: (**a**) La_2_Zr_2_O_7_; (**b**) HEC-0; (**c**) HEC-0.2; (**d**) HEC-0.4; (**e**) HEC-0.5.

Moreover, the corrosion products and their morphologies were further analyzed, in conjunction with the semi-quantitative EDS analysis presented in Figure 6. Following 8 h of CMAS corrosion, the reaction layer predominantly consists of light gray rod-shaped crystals and spherical grains. The rod-shaped crystals exhibit a high concentration of silicon (Si) and a low concentration of zirconium (Zr), with the presence of calcium (Ca) and rare earth (RE) elements, and trace amounts of magnesium (Mg) and aluminum (Al), which can be identified as apatite (Ca*_x_*RE_10−*x*_(SiO_4_)_6_O_3−*x*/2_, 2 ≤ *x* ≤ 6). On the other hand, the spherical corrosion products are richer in Zr and RE. The content of Ca and Si elements is significantly reduced in the rod-shaped corrosion products, while the spherical grains can be regarded as ZrO_2_ doped with RE and Ca. The solubility of rare earth elements in corrosion products varies depending on their ionic radii. Taking the example of the Sm, Gd, Dy, and Er rare earth elements, the content of rod-shaped corrosion products follows the order: Sm > Gd > Dy > Er, while for spherical corrosion products, it is Er > Dy > Gd > Sm. This indicates that rare earth elements with a larger ionic radius tend to dissolve in apatite, whereas those with a smaller ionic radius tend to dissolve in ZrO_2_.

**Figure 7:**
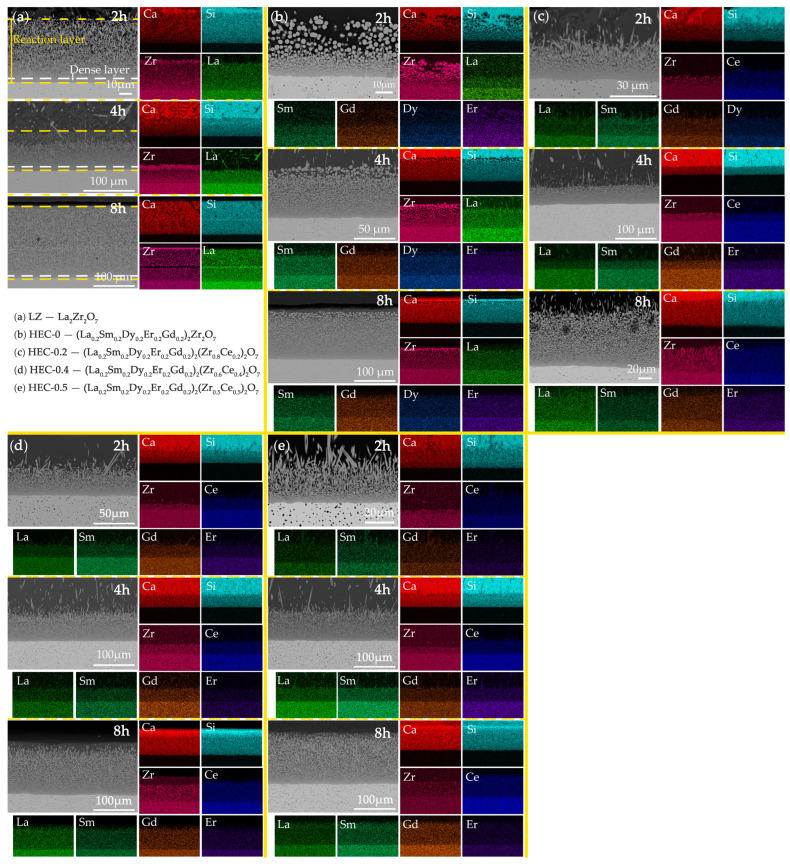
Cross-sectional backscattered electron morphology and element distribution map of the five rare earth zirconate bulks after different durations of CMAS corrosion: 2 h; 4 h; and 8 h. (**a**) La_2_Zr_2_O_7_; (**b**) HEC-0; (**c**) HEC-0.2; (**d**) HEC-0.4; (**e**) HEC-0.5.

To facilitate a clearer observation of the variation in corrosion layer thickness and product distribution, Figure 7 displays the cross-sectional backscattered electron morphology and an element distribution map of the five rare earth zirconate bulks after different durations of CMAS corrosion: 2 h; 4 h; and 8 h. It is found that the cross-section morphology can be divided into three parts: the zirconate block, the residual CMAS, and the corrosion reaction layer between the block and CMAS. The latter consists of a dense layer below and a loose reaction layer above, which consists of spherical and rod-shaped crystals. The uninfiltrated CMAS regions, characterized by the highest intensity of Ca and Si elements in the EDS analysis or dark regions of backscattered electron morphology, exhibit a distribution pattern of large-sized apatite products. The entire reaction layer consists of Zr, rare earth elements, Ca, and Si, with the boundary between the reaction layer and the matrix distinguishable by the dark regions in the Ca and Si element mapping. Similarly, the boundary between the reaction layer and residual CMAS can be identified through the polarized Zr element. Throughout the corrosion process, including at the 8 h mark, no detectable concentrations of Mg and Al were observed. The growth direction of apatite is approximately perpendicular to the interface between the reaction layer and the substrate, exhibiting a high degree of consistency in size within the reaction layer.

**Figure 8:**
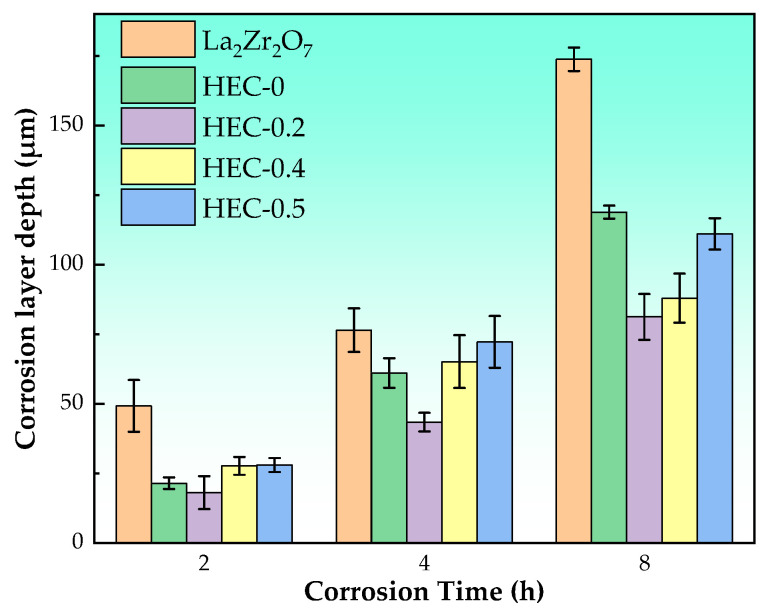
Corrosion layer depth and corrosion time of five rare earth zirconates.

The thickness of the corrosion layer was determined based on SEM images (Figure 7), where a distinct stratification consisting of a compact lower layer and a loosely adhered upper layer was clearly observed. Equation (1) was employed to calculate the corrosion layer thickness at each position within the sample [31].

(1) $$h=\frac{1}{x}\sum_{i=1}^{x}d_{i}$$

where $d_{i}$ is the thickness of the corrosion layer at each position, and a minimum of 20 positions for each sample are analyzed. The corresponding results are presented in Figure 8. The thickness of the corrosion layer increases over time. Among all samples, La_2_Zr_2_O_7_ exhibits the largest corrosion layer thickness under all corrosion durations. High-entropy samples show a significantly reduced corrosion thickness compared to La_2_Zr_2_O_7_, which can be attributed to the hysteretic diffusion effect induced by the high-entropy strategy. The high-entropy sample facilitates the formation of a stable apatite phase, thereby enhancing the corrosion layer’s stability and effectively retarding the infiltration of CMAS melt. The overall thickness of the corrosion layer exhibits a trend of initially decreasing and then increasing with the increase in Ce content at position B. Notably, the HEC-0.2 sample consistently displays the lowest corrosion layer thickness at each corrosion duration.

## 4. Discussion

### 4.1. Reaction Product

According to the above results, the chemical equation of the reaction between rare earth zirconate ceramics and CMAS melt can be written as

$$3{\mathrm{La}}_{2}{\mathrm{Zr}}_{2}O_{7}+6{\mathrm{SiO}}_{2}+4\mathrm{CaO}\to {\mathrm{Ca}}_{4}{\mathrm{La}}_{6}{\left({\mathrm{SiO}}_{4}\right)}_{6}O+6{\mathrm{ZrO}}_{2}$$

The crystal structure of apatite can be written as $X_{4}^{\prime }X_{6}^{\prime \prime }{\left(SiO_{4}\right)}_{6}O_{n}$ with a hexagonal *P*6_3_*/m* space group [32,33]. The $X^{\prime }$ site is coordinated with nine oxygen ions, usually occupied by Ca ions; the $X^{\prime \prime }$ site is coordinated with seven oxygen ions, usually occupied by RE ions [34,35]. For high-entropy rare earth zirconates, the situation is different. Due to the presence of multiple rare earth ions with varying ionic radii, some rare earth ions have similar ionic radii to Ca ions, leading to a situation where Ca ions occupy the $X^{\prime \prime }$ site and RE ions occupy the $X^{\prime }$ site. Therefore, the chemical equation needs to be rewritten as (2 ≤ x ≤ 6).

$$\frac{10-x}{2}{\left(5{\mathrm{RE}}_{0.2}\right)}_{2}{\mathrm{Zr}}_{2}O_{7}+6{\mathrm{SiO}}_{2}+x\mathrm{CaO}\to {\mathrm{Ca}}_{x}{\left(5{\mathrm{RE}}_{0.2}\right)}_{10-x}{\left({\mathrm{SiO}}_{4}\right)}_{6}O_{3-\frac{x}{2}}+(10-x){\mathrm{ZrO}}_{2}$$

### 4.2. Mechanism of High-Temperature CMAS Corrosion with Rare Earth Zirconates

**Figure 9:**
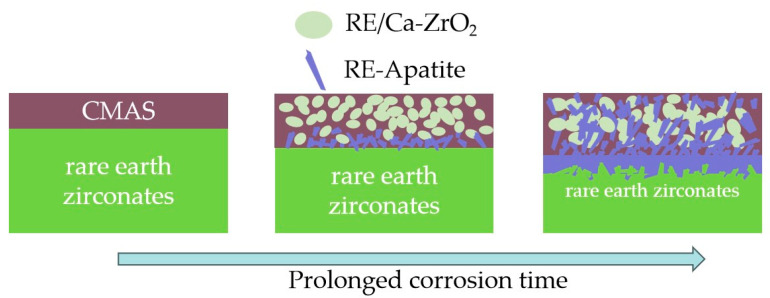
Schematic diagram of high-temperature CMAS corrosion mechanism.

The schematic diagram in Figure 9 illustrates the high-temperature corrosion mechanism of rare earth zirconates for CMAS. The CMAS powder undergoes melting and surface spreading at a temperature of 1300 °C, effectively infiltrating the matrix by permeating through pores and microcracks. Among the lanthanide rare earth elements, those with lower atomic numbers (La, Nd, Sm, Eu, Gd) exhibit smaller differences in atomic radius compared to Ca^2+^, thereby facilitating the formation of the apatite phase. As the melt infiltrates the surface and dissolution intensifies, once a specific concentration of rare earth elements is reached, Ca and Si in CMAS are consumed to form an apatite phase which begins to crystallize. The concentration of rare earth elements decreases, causing residual Zr and rare earth elements to polarize into spherical ZrO_2_ that remains in situ. With the diffusion of Ca and Si into the bulk material, apatite and ZrO_2_ form a dense layer between the matrix and reaction layer. Initially, apatite mainly accumulates near the dense layer, while residual ZrO_2_ remains above it. The entire dense layer moves downward simultaneously with diffusion, leaving behind a loose reactive layer. Due to the downward diffusion process, apatite tends to grow perpendicular to the interface between the reaction layer and the substrate. With a prolonged reaction time, there is only a slight increase in the thickness of the dense layer; however, it continues to diffuse downwards until all of the melt is consumed.

### 4.3. Effect of High-Entropy Strategy on Corrosion Resistance

**Figure 10:**
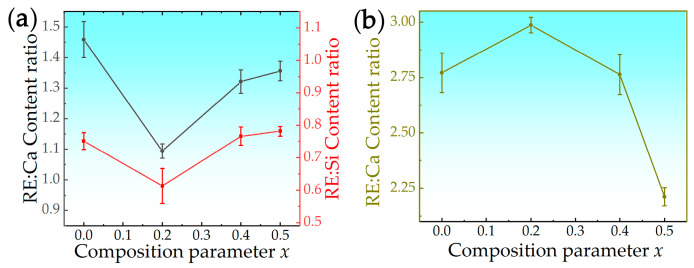
The ratio of (**a**) RE:Ca and RE:Si in apatite, and (**b**) RE:Ca in ZrO_2_, after 4 h corrosion of high-entropy (La_0.2_Sm_0.2_Dy_0.2_Er_0.2_Gd_0.2_)_2_(Zr_1−*x*_Ce*_x_*)_2_O_7_ series.

The analysis of Figure 8 leads to the conclusion that the high-entropy strategy significantly enhances the corrosion resistance of rare earth zirconates. Furthermore, a more distinct manifestation of the impact of high entropy can be observed by examining the alteration in the RE:Ca and RE:Si ratios, as depicted in Figure 10. The production of apatite requires a significant amount of Si, as well as some Ca and rare earth elements, whereas the production of ZrO_2_ solely necessitates Ca and rare earth elements. When the Ce addition amount is x = 0.2, there is a significant decrease in the RE:Ca and RE:Si ratios in apatite, indicating that the incorporation of rare earth elements into apatite from CMAS in HEC-0.2 becomes more challenging compared to other samples. The increase in Ce (x = 0.2), despite being a B-position element in rare earth zirconate, leads to an elevation in the entropy of the resulting rare earth calcium silicate oxyapatite (Ca*_x_*RE_10−*x*_(SiO_4_)_6_O_3−*x*/2_, 2 ≤ x ≤ 6) [36,37]. Consequently, this significantly hampers the mutual diffusion rate between rare earth elements and Ca and Si, causing a sharp decline in the RE:Ca and RE:Si ratios. The suppression of diffusion evidently enhances the corrosion resistance of rare earth zirconates. The similarity in the contents of different rare earth elements in apatite can also be observed in Figure 6c.

However, with the further increase in Ce doping, the entropy-stable distribution of rare earth elements is disrupted. Due to Ce’s smaller ionic radius, it facilitates the substitution of Ca^2+^ and enhances apatite formation more significantly. The resulting apatite also exhibits a higher concentration of Ce compared to other rare earth elements in Figure 6d,e. The RE:Ca ratio in ZrO_2_ exhibits a contrasting trend compared to that observed in apatite.

## 5. Conclusions

In this study, we have observed that the resistance of high-entropy rare earth zirconates to CMAS corrosion is significantly superior compared to single rare earth zirconates. Among them, the high-entropy component HEC-0.2 exhibits the highest level of corrosion resistance due to its ability to maximize the hysteretic diffusion effect of high entropy. Rare earth elements with larger ionic radii tend to dissolve in apatite, while those with smaller ionic radii tend to dissolve in ZrO_2_.

In conclusion, employing a high-entropy strategy proves effective in enhancing the resistance of rare earth zirconates against CMAS penetration and corrosion, making them promising candidates for TBC materials.

## Figures and Tables

**Table 1:** Composition as well as powder calcination and bulk sintering processes.

Composition	Abbreviation	Calcination	Sintering
(La_0.2_Sm_0.2_Dy_0.2_Er_0.2_Gd_0.2_)_2_Zr_2_O_7_	HEC-0	1400 °C 6 h	1650 °C 10 h
(La_0.2_Sm_0.2_Dy_0.2_Er_0.2_Gd_0.2_)_2_(Zr_0.8_Ce_0.2_)_2_O_7_	HEC-0.2		
(La_0.2_Sm_0.2_Dy_0.2_Er_0.2_Gd_0.2_)_2_(Zr_0.6_Ce_0.4_)_2_O_7_	HEC-0.4		
(La_0.2_Sm_0.2_Dy_0.2_Er_0.2_Gd_0.2_)_2_(Zr_0.5_Ce_0.5_)_2_O_7_	HEC-0.5		
La_2_Zr_2_O_7_	-		

**Table 2:** Theoretical density, measured density, and relative density of samples.

Abbreviation	Measured Density (g∙cm^−3^)	Theoretical Density (g∙cm^−3^)	Relative Density (%)
HEC-0	6.698	6.851	97.77
HEC-0.2	6.765	6.971	97.04
HEC-0.4	6.713	7.087	94.71
HEC-0.5	6.732	7.144	94.23
La_2_Zr_2_O_7_	5.833	6.031	96.72

## Data Availability

The original contributions presented in this study are included in the article. Further inquiries can be directed to the corresponding author.
